# Functional and clinical data of Best vitelliform macular dystrophy patients with mutations in the *BEST1* gene

**Published:** 2009-12-31

**Authors:** Giuseppe Querques, Jennyfer Zerbib, Rossana Santacroce, Maurizio Margaglione, Nathalie Delphin, Jean-Michel Rozet, Josseline Kaplan, Domenico Martinelli, Nicola Delle Noci, Gisèle Soubrane, Eric H. Souied

**Affiliations:** 1 Department of Ophthalmology, Hopital Intercommunal de Creteil, University Paris XII, Paris, France; 2 Department of Ophthalmology, Ospedali Riuniti, University of Foggia, Foggia, Italy; 3 Department of Genetics, Ospedali Riuniti, University of Foggia, Foggia, Italy; 4 Department of Genetics, Necker Hospital, University Paris V, Paris, France; 5 Department of Hygiene, Policlinico di Bari, University of Bari, Bari, Italy; 6 Unite Fonctionnelle de Recherche Clinique, Creteil, France

## Abstract

**Purpose:**

To analyze functional and clinical data of Best vitelliform macular dystrophy (VMD) patients with mutations in the *BEST1* gene.

**Methods:**

Best VMD patients with *BEST1* mutations were evaluated prospectively regarding age, age of onset, best-corrected visual acuity (BCVA), fundus autofluorescence, fluorescein angiography, optical coherence tomography, and electro-oculography. Mutations in *BEST1* were established by direct sequencing.

**Results:**

Forty-six eyes of 23 patients (10 male, 13 female) were included in the study. We identified nine different *BEST1* mutations (3/9 novel), in ten unrelated families. The age of patients ranged between 3 and 75 years; age of onset varied between 2 and 67 years. BCVA ranged between 20/20 and 20/200. On the basis of fundus biomicroscopy with direct illumination, using one widely accepted classification, the macular lesions could be counted as follows: 1. no lesion (normal fovea): eight eyes, five patients carrying a mutation on the *BEST1* gene; 2. previtelliform lesions: six eyes, three affected patients; 3. vitelliform lesions: four eyes, two affected patients; 4. pseudohypopyon: three eyes, three affected patients; 5. vitelliruptive lesions (scrambled egg aspect with dispersion of the vitelliform material without sign of atrophy or fibrosis): ten eyes, six affected patients; 6. atrophic lesions (atrophy with or without residual dispersed material): seven eyes, five patients; 7. fibrotic lesions: eight eyes, five patients. Two patients presented unilateral Best VMD. Both eyes of two patients presented multifocal Best VMD features on fundus examination. Six eyes of four patients have been treated for choroidal neovascularization by thermic photocoagulation [one eye], photodynamic therapy [three eyes], and intravitreal ranibizumab injection [two eyes]. Comparison of interfamilial and intrafamilial clinical data between patients did not reveal differences in age, BCVA, and stage of the disease as evaluated by fundus autofluorescence, fluorescein angiography, and optical coherence tomography (p>0.05). Mean BCVA impairment showed a statistically significant correlation to a more advanced stage of the disease (p<0.001).

**Conclusions:**

*BEST1* mutations were not correlated with the severity of the functional and clinical data in the Best VMD patients examined.

## Introduction

Vitelliform macular dystrophy (VMD) was first described by Friedrich Best in 1905 with a complete description of the various stages of the disease from eight related individuals [1]. VMD (OMIM 153700), also called Best disease, has an autosomal dominant pattern of inheritance but with variable expressivity. The gene involved in Best VMD, called *BEST1*, has been mapped on chromosome 11q12-q13, cloned, and sequenced [2]. The 68-kDa protein encoded by the *BEST1* gene, named bestrophin-1 [3], is localized to the basolateral plasma membrane of the retinal pigment epithelium (RPE) and appears to exhibit properties of Ca^2+^-activated Cl^−^ channels [4]. More than 100 disease-causing mutations in *BEST1* have been reported (HGMD), with nearly all of those causing Best VMD affecting single amino acids at one of 66 different positions in bestrophin-1. The onset of Best VMD is variable, having a bimodal distribution with one maximum peak before puberty and a second following puberty and extending through the fifth decade of life [5]. Heterozygous mutations in *BEST1* may also cause the adult form of VMD, autosomal recessive bestrophinopathies, other autosomal dominant bestrophinopathies, and rare vitreoretinochoroidopathy.

Best VMD is a clinically heterogeneous and pleomorphic disease; usually it begins with symptoms of metamorphopsia, blurred vision, and a decrease of central vision. Most cases have a solitary lesion in the macula; others have multifocal vitelliform lesions [6,7], which are mostly confined to the posterior pole. Five stages have been described, based on fundus examination [8]: the previtelliform stage (normal macula or subtle RPE alterations), the vitelliform stage (well circumscribed 0.5- to 2-disc-diameter “egg-yolk” lesion), the pseudohypopyon stage (the yellow material accumulated inferiorly), the vitelliruptive stage (partial resorption of the material, scrambled-egg lesion), and the atrophic stage (final macular atrophy). An aspect of fibrosis (elevated changes from white to yellowish) can also be observed as an optional way of evolution of VMD. This cicatricial aspect can appear with or without occurrence of choroidal neovascularization (CNV). There is a controversy about the chronological order of the stages. According to some authors, the sequence of the pseudohypopion/vitelliruptive stages may be reversed.

Abnormal electro-oculogram (EOG) [9,10], with a reduced or nondetectable light-peak to dark-trough ratio (≤1.55), combined with a normal clinical electroretinogram (ERG) [11], a blockage effect by vitelliform material on fluorescein angiography [12], and autofluorescence from the vitelliform lesions^7^ are helpful for diagnosis. Spaide and associates illustrated by optical coherence tomography (OCT) that the yellow vitelliform accumulates in the subretinal space and on the outer retinal surface [13]. We recently reported on the high-definition spectral domain optical coherence tomography (HD-OCT; OCT 4000 Cirrus; Humphrey-Zeiss, San Leandro, CA) findings in all the progressive stages of the disease, including the previtelliform (preclinical) stage [14,15].

Our purpose in this study was to analyze the functional and clinical data in Best VMD patients, issuing primarily from one single family, according to the mutations in the *BEST1* gene.

## Methods

Best VMD patients and relatives that presented consecutively at the Créteil University Eye Clinic, Creteil, France, and at the Foggia University Eye Clinic, Foggia, Italy were included in this prospective study. The clinical diagnosis, based on one or multiple subfoveal vitelliform lesions in at least one eye, was confirmed by two observers (GQ, EHS). At least one affected individual from each family was diagnosed by both EOG and fundus examination. Informed consent was obtained according to approved protocols of the Paris XII University and Foggia University Institutional Review Boards, in agreement with the Declaration of Helsinki. The patients were evaluated based on age and age of onset (age at initial examination for visual impairment), and all underwent a complete ophthalmologic examination, including assessment of best-corrected visual acuity (BCVA) measured at 4 m with standard Early Treatment Diabetic Retinopathy Study charts, fundus biomicroscopy, color photography of the fundus (Topcon TRC-50 retinal camera, Tokyo, Japan), fundus autofluorescence (FAF) frames (Heidelberg Retina Angiograph II, Heidelberg Engineering, Heidelberg, Germany), and red-free and fluorescein angiography (FA) frames (Topcon TRC-50 retinal camera, Tokyo, Japan; Heidelberg Retina Angiograph II, Heidelberg Engineering). Recordings of EOG and ERG (in selected cases) were done according to the International Society for Clinical Electrophysiology of Vision standard [16,17]. OCT examination was performed with time domain OCT (OCT 3000 Stratus, Humphrey-Zeiss) and spectral domain OCT (HD-OCT, OCT 4000 Cirrus, Humphrey-Zeiss). All scans were positioned within the macular area and throughout the vitelliform lesions, based on color fundus photography and FAF. For each scan the shape and reflectivity of the material, its location, the reflectivity and appearance of the RPE, and retinal changes were specified. The diagnosis of Best VMD was based on the presence of large vitelliform or vitelliruptive lesions and a reduced light rise in the EOG, with or without a positive family history of the disease.

All patients were screened for mutations in the *BEST1* gene by direct sequencing. Genomic DNA was submitted to standard PCR, using intronic primers designed to flank the coding exons (2–11) and exon–intron boundaries of the *BEST1* gene (primer sequences and PCR conditions are listed in Table 1). Amplified products were directly sequenced without preliminary purification using the Big Dye Terminator Cycle Sequencing kit v3.1 (Applied Biosystems, Foster City, CA). Sequenced products were purified by exclusion chromatography (Sephadex G50; Sigma-Aldrich, Saint Louis, MO), submitted to electrophoresis on an ABI 3130 automated sequencer (Applied Biosystems, Foster City, CA), and data were analyzed using Sequencing Analysis v5.2 Software (Applied Biosystems). All exons were screened in all probands. Segregation of the mutations with the disease phenotype was established by using the available family members. The pathogenicity of unreported nucleotide changes was assessed by i) studying 96 unrelated control individuals (control group) matched for origin with no personal or familial history of macular degeneration or retinal dystrophy, and ii) applying the Polyphen (Harvard University, Boston, MA) program, which predicts possible impact (benign, possibly damaging, probably damaging or unknown) of an amino acid substitution on the structure and function of human proteins (as previously reported by Ramensky et al. [18]).

**Table 1:** Primer sequences and PCR conditions.

**Exon**	**Sequence of primers**	**Number of cycles**	**Annealing temperature (°C)**
2	F-AGTCTCAGCCATCTCCTCGC	35	62
	R-TGGCCTGTCTGGAGCCTG		
3	F-GGGACAGTCTCAGCCATCTC	35	60
	R-CAGCTCCTCGTAGTCCTCC		
4	F-AGAAAGCTGGAGGAGCCG	35	60
	R-GCGGCAGCCCTGTCTGTAC		
5	F-GGGGCAGGTGGTGTTCAGA	35	60
	R-GGCAGCCTCACCAGCCTAG		
6	F-GGGCAGGTGGTGTTCAGA	35	60
	R-CCTTGGTCCTTCTAGCCTCAG		
7	F-CATCCTGATTTCAGGGTTCC	35	60
	R-CTCTGGCCATGCCTCCAG		
8	F-AGCTGAGGTTTAAAGGGGGA	35	60
	R-TCTCTTTGGGTCCACTTTGG		
9	F-ACATACAAGGTCCTGCCTGG	35	60
	R-GCATTAACTAGTGCTATTCTAAGTTCC		
10 A	F-GGTGTTGGTCCTTTGTCCAC	35	60
	R-CTCTGGCATATCCGTCAGGT		
10 B	F-CTTCAAGTCTGCCCCACTGT	35	60
	R-TAGGCTCAGAGCAAGGGAAG		
11	F-CATTTTGGTATTTGAAATGAAGG	35	60
	R-CCATTTGATTCAGGCTGTTG		

Statistical analyses were performed using STATA 10 MP (StataCorp LP, College Station, TX) for MacOs X. Serial interfamilial and intrafamilial comparisons of specific *BEST1* mutations and expressivity with respect to age, BCVA converted to the logarithm of the minimum angle of resolution (logMAR), and stage of the disease were performed using the ANOVA (ANOVA) test. The chosen level of statistical significance was p<0.05. Of note, most of the Best VMD patients analyzed for this study issued from one single family, and it is probably a major limitation of any statistical analysis to propose a severity scaling.

## Results

### Genetic analysis

**Figure 1:**
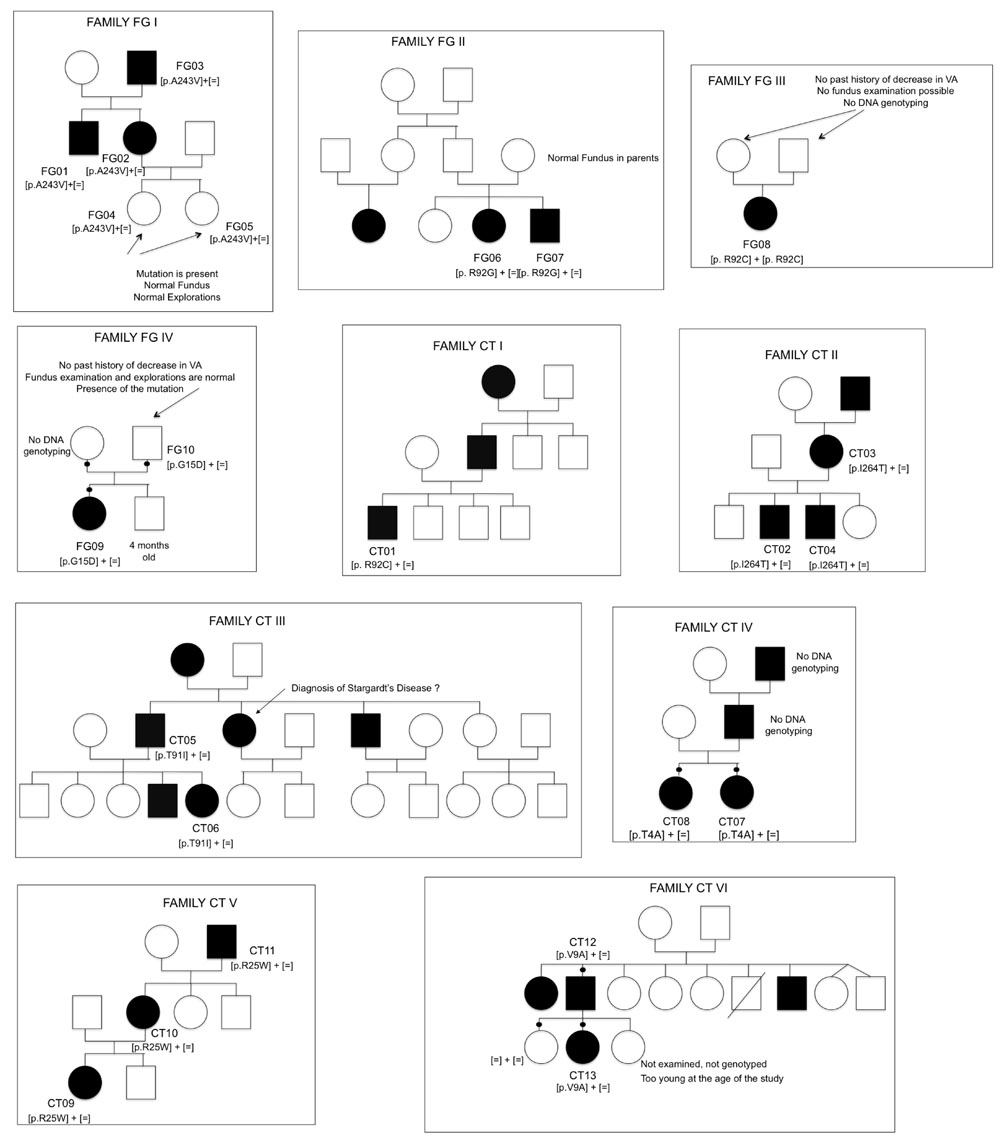
Pedigrees of the families studied and segregation of the VMD2 mutant alleles. White circles represent unaffected females, filled circles affected females, white squares unaffected males, and filled squares affected males. Deceased individuals are shown with a slanting line across the symbol.

**Figure 2:**
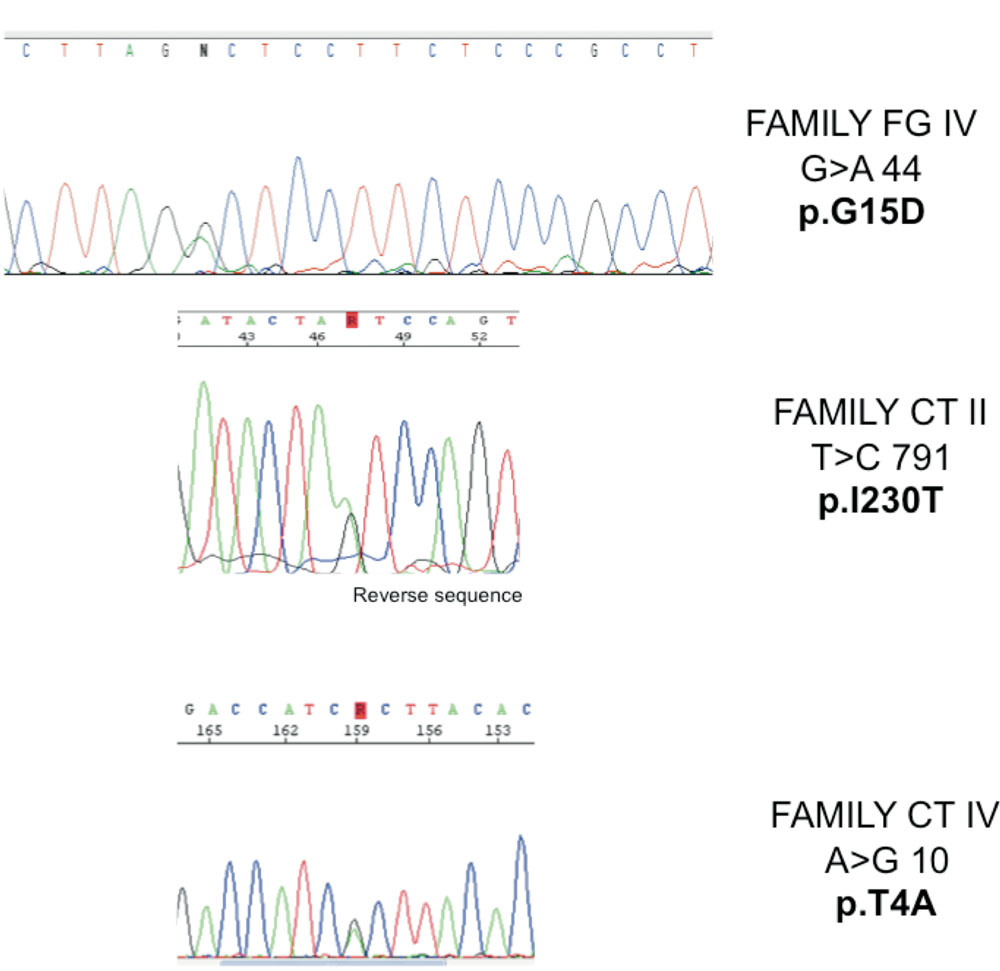
Electropherograms of the three novel *BEST1* mutations. These electrophoregrams show an heterozygous peak GA at position 44 responsible for a p.G15D mutation in family FG IV, an heterozygous peak TC at position 791 responsible for a p.I230T mutation in family CT II, and an heteozygous peak AG at position 10 responsible for a p.T4A mutation in family CT IV.

**Figure 3:**
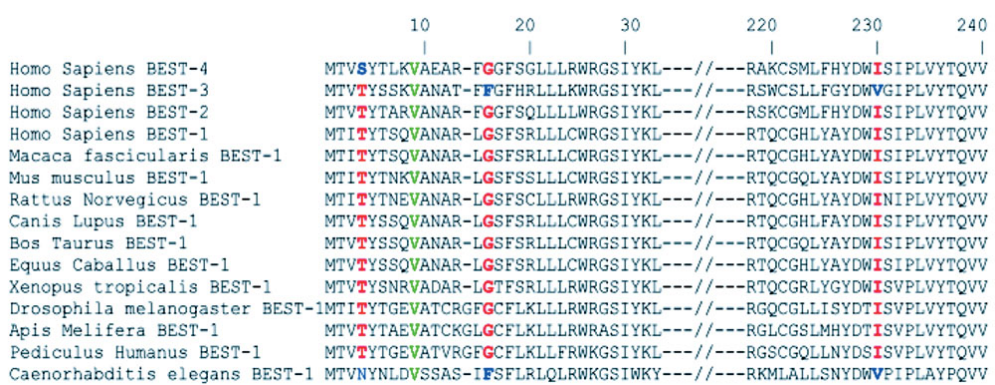
Protein sequence alignments via Protein Basic Local Alignment Search Tool (BLASTP) of the regions of the human proteins of the BEST family (BEST1–4) and of the BEST1 proteins containing the p.T4A, p.G15D, and p.I230T novel mutations. The residues at position 4, 15, and 230 are highly conserved from mammals to flies as well as in two-thirds of the human BEST proteins. Interestingly, when nonconserved, the amino acids are replaced by residues of the same classes (neutral polar threonine at position 4 is changed to neutral polar asparagine and serine in human BEST4 and worm BEST1 proteins, respectively; nonpolar uncharged glycine at position 15 is changed to uncharged nonpolar phenylalanine in the human BEST3 and worm BEST1 sequences, respectively; neutral nonpolar isoleucine at position 230 is changed to valine in the human BEST3 and worm BEST1 proteins). Interestingly, the three novel BEST1 mutations reported here are expected to change the polarity and/or the charge of the protein. The p.T4A mutation changes a polar to a nonpolar amino acid, while the p.G15D and I230T mutations change nonpolar uncharged residues to polar acidic (aspartic acid) and neutral nonpolar (threonine) residues, respectively.

The screening of the 11 exons encoding *BEST1* in ten unrelated families (4/10 from Italy; 6/10 from France; Figure 1) resulted in the identification of nine different missense mutations clustered in exons 2, 4, and 7 (Table 2). Six out of the nine mutations have been previously reported to be common Best VMD mutations (p.A243V, p.R92G, p. R92C, p.T91I, p.R25W, p.V9A). The remaining three changes have not been reported elsewhere (p.T4A, p.G15D, p.I230T; Figure 2) and were absent from 192 control chromosomes. Interspecies Basic Local Alignment Search Tool (BLAST) alignments showed that residues at positions 4, 15, and 230 are conserved in vertebrate and invertebrate species (Figure 3). Simulation for functional changes by a structure homology-based method using the Polyphen program resulted in classifying the p.T4A and p.G15D changes as possibly damaging (position-specific independent counts, PSIC=1.847 and 1.936, respectively, and the p.I230T substitution as probably damaging (I230T; PSIC=2.181).

**Table 2:** Summary of clinical findings and probands *BEST1* mutations.

**Patient**	**Mutation**	**Position**	**Missense effect**	**Age-Gender**	**Age of onset**	**Lesion type RE**	**Lesion type LE**	**BCVA RE**	**BCVA LE**	**Complications**
FG01 (Family FG I)	C>T728 heterozygous	exon 7	A243V	49-M	41-	atrophy	atrophy	20/125	20/160	-
FG02 (Family FG I)	C>T728 heterozygous	exon 7	A243V	45-F	37-	vitelliruptive	vitelliruptive	20/25	20/25	-
FG03 (Family FG I)	C>T728 heterozygous	exon 7	A243V	75-M	67-	pseudohypopion	vitelliruptive	20/50	20/125	-
FG04 (Family FG I)	C>T728 heterozygous	exon 7	A243V	13-F	-	none	none	20/20	20/20	-
FG05 (Family FG I)	C>T728 heterozygous	exon 7	A243V	17-F	-	none	none	20/20	20/20	-
FG06 (Family FG II)	G>A275 heterozygous	exon 4	R92G	16-F	11-	fibrosis	fibrosis	20/160	20/160	CNV RLE
FG07 (Family FG II)	G>A275 heterozygous	exon 4	R92G	3-M	2-	vitelliform	vitelliform	20/32	20/32	-
FG08 (Family FG III)	C>T 274 homozygous	exon 4	R92C	16-F	15-	vitelliruptive+multifocal	vitelliruptive+multifocal	20/32	20/40	-
FG09 (Family FG IV)	G>A44 heterozygous	exon 2	G15D	3-F	2-	vitelliform	vitelliform	20/25	20/25	-
FG10 (Family FG IV)	G>A44 heterozygous	exon 2	G15D	30-M	-	none	none	20/20	20/20	-
CT01 (Family CT I)	C>T274 heterozygous	exon 4	R92C	14-M	8-	fibrosis	fibrosis	20/50	20/40	CNV RLE
CT02 (Family CT II)	T>C791 heterozygous	exon 7	I230T	11-M	10-	pre-vitelliform	pre-vitelliform	20/20	20/25	-
CT03 (Family CT II)	T>C791 heterozygous	exon 7	I230T	42-F	41-	pre-vitelliform+multifocal	pre-vitelliform+multifocal	20/32	20/25	-
CT04 (Family CT II)	T>C791 heterozygous	exon 7	I230T	9-M	6-	vitelliruptive	vitelliruptive	20/125	20/125	-
CT05 (Family CT III)	C>T272 heterozygous	exon 4	T91I	44-M	36-	atrophy	atrophy	20/125	20/40	-
CT06 (Family CT III)	C>T272 heterozygous	exon 4	T91I	19-F	11-	fibrosis	fibrosis	20/200	20/40	CNV RE
CT07 (Family CT IV)	A>G10 heterozygous	exon 2	T4A	27-F	20-	atrophy	none	20/50	20/25	-
CT08 (Family CT IV)	A>G10 heterozygous	exon 2	T4A	23-F	16-	pseudohypopion	atrophy	20/32	20/50	CNV LE
CT09 (Family CT V)	C>T73 heterozygous	exon 2	R25W	10-F	9	vitelliruptive	fibrosis	20/20	20/200	-
CT10 (Family CT V)	C>T73 heterozygous	exon 2	R25W	36-F	30-	vitelliruptive	vitelliruptive	20/63	20/63	-
CT11 (Family CT V)	C>T73 heterozygous	exon 2	R25W	70-M	60-	pseudohypopion	none	20/50	20/20	-
CT12 (Family CT VI)	T>C26 heterozygous	exon 2	V9A	44-M	7-	atrophy	fibrosis	20/50	20/200	-
CT13 (Family CT VI)	T>C26 heterozygous	exon 2	V9A	12-F	12-	pre-vitelliform	pre-vitelliform	20/20	20/20	-

Abbreviations: M represents male; F represents female; RE represents right eye; LE represents left eye; BCVA represents best corrected visual acuity; CNV represents choroidal neovascularization.

**Figure 4:**
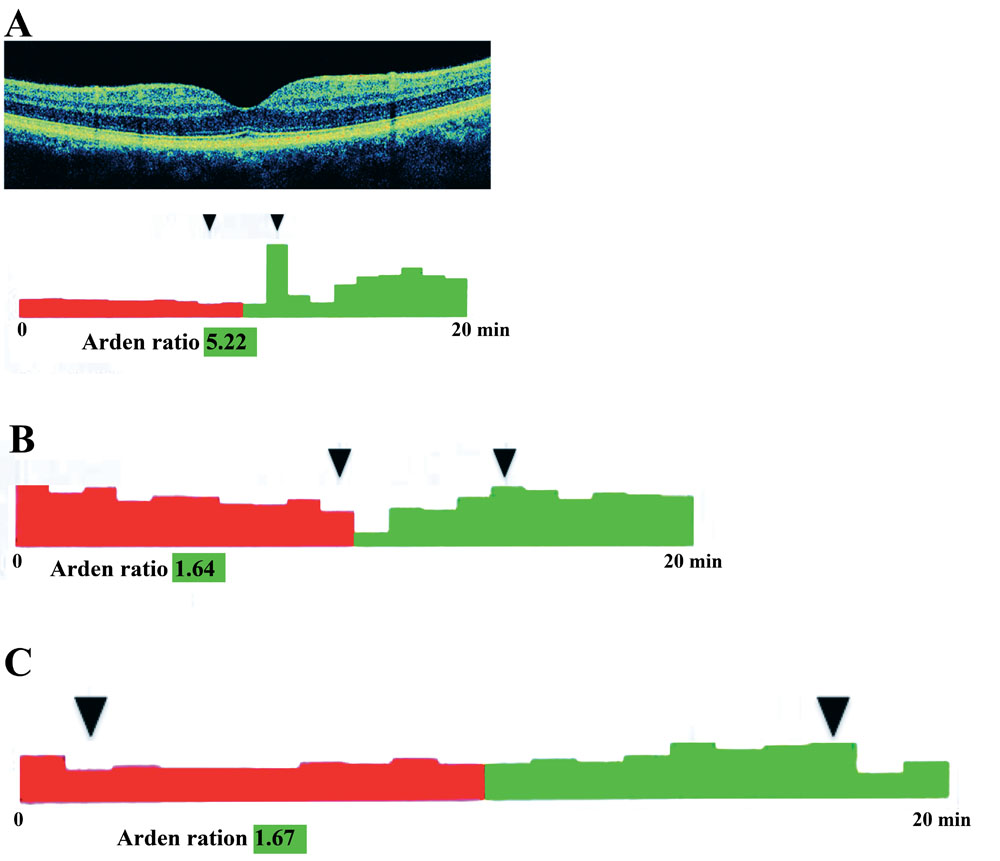
Spectral domain high-definition optical coherence tomography and electro-oculogram findings of patient FG10, patient FG05 and patient FG04. Spectral domain high-definition optical coherence tomography scan of the right eye of patient FG10 (**A**, upper panel**)** shows normal macular findings. Electro-oculogram of the same eye (**A**, bottom panel) shows the light-peak saccade not uniform, being the light-peak to dark-trough ratio overall normal (>1.55). Electro-oculograms of the right eye of patient FG05 (**B**), and of the left eye patient FG04 (**C**), show normal light-peak to dark-trough ratio (>1.55).

Segregation analyses were performed when possible. In all families but one, available affected patients were shown to be heterozygous for the mutation. In family FGIII, the affected patient FG08 was apparently homozygous for the p.R92C mutation. Parental DNA samples were unavailable to determine between homozygosity and deletion at the *BEST1* locus. This apparently homozygous p.R92C finding could clearly be homozygous or could reflect a combination between a point mutation in one allele and a deletion in the other allele. However, homozygosity is probable as both parents were born in the same village of the Puglia region in Italy. Finally, that three unaffected individuals harbored the pathogenic mutation identified in their families (patients FG04, FG05, and FG10; Figure 4).

Finally, our data add another example of amino acid residues that produce Best VMD when mutated to different amino acids: arginine at position 92 was substituted by a glycine in patient FG06 and patient FG07 (FAMILY FG II) or by a cysteine in patient FG08 (FAMILY FG III) and patient CT01 (FAMILY CT I).

### Functional and clinical data

We examined 46 eyes of 23 patients harboring *BEST1* mutations (10 male, 13 female). Eighteen had Best VMD, two presented with multifocal Best VMD, and three were asymptomatic (Table 1). The mean age of patients was 27.30 ±20.09 years. Age of onset varied between 2 and 67 years (median=13.5). BCVA ranged between 20/20 and 20/200 (median, 20/35). All affected patients had bilateral lesions except two unrelated patients who had a unilateral lesion (CT07 aged 27 years and CT05 aged 70 years).

On the basis of fundus biomicroscopy with direct illumination (performed by two expert retinal physicians [G.Q., E.H.S.]) and using one widely accepted classification, the macular lesions could be counted as follows: 1. no lesion (normal fovea): eight eyes, five patients carrying a mutation on the *BEST1* gene; 2. previtelliform lesions: six eyes, three affected patients; 3. vitelliform lesions: four eyes, two affected patients; 4. pseudohypopyon: three eyes, three affected patients; 5. vitelliruptive lesions (scrambled egg aspect with dispersion of the vitelliform material without sign of atrophy or fibrosis): ten eyes, six affected patients; 6. atrophic lesions (atrophy with or without residual dispersed material): seven eyes, five patients; 7. fibrotic lesions: eight eyes, five patients.

Early stage lesions were characterized by the accumulation of yellowish material within the macula and within and/or outside the macular area, giving an aspect of foveal granularity (previtelliform lesions) or a typical well circumscribed yellow “egg yolk” (vitelliform lesion). This material was highly autofluorescent. On OCT scans it appeared as a hyper-reflective dome-shaped lesion located between the hyporeflective outer nuclear layer and the hyper-reflective RPE layer.

Later stages included pseudohypopyon and vitelliruptive (scrambled egg aspect with dispersion of the vitelliform material without sign of atrophy or fibrosis) lesions that were characterized by partial/complete resorption of the yellowish material, which was replaced by a fluid component showing no increased fluorescence on FAF and reflectivity on OCT examination. These stages were characterized by loss of continuity and centrifugal and downward movement of the lipofuscin-like material.

Late lesions were characterized by partial/complete atrophy (with or without residual dispersed material) or fibrosis (with no detectable active CNV) within the area previously occupied by the yellowish material. FA showed both masking effects, from accumulation of material, and transmission defects, from resorption of material, with passive leakage. The EOG showed an abnormal light-peak to dark-trough ratio (<1.55) in all affected eyes.

Even with the same mutation, the age of onset and the disease progression (stage of the disease and visual function) were highly variable interfamilially and intrafamilially. The heterozygous p.R92G and p.G15D mutations accounted for the earliest disease manifestations in our series (at 2 years of age, patient FG07, FAMILY FGII and patient FG09, FAMILY FG IV, respectively) or either a later onset (at the age of 11 years for patient FG06, FAMILY FGII [p.R92G]) or even no disease manifestation (at the age of 30 years for patient FG10 FAMILY FG IV [p.G15D]), respectively.

On the other hand, the heterozygous p.R92G, p.R92C, p.T91I, and p.T4A mutations resulted in CNV development (which was treated by photodynamic therapy [both eyes of FG06, right eye of CT01], intravitreal ranibizumab injection [left eye of CT01, right eye of CT06], and thermic photocoagulation [extrafoveal CNV, left eye of CT08]).

Three cases harboring heterozygous *BEST1* mutations in two families showed normal fundus findings, OCT, and EOG (FG04 and FG05 [p.A243V], FAMILY FG I and FG10 [p.G15D], Family FG IV). Two unrelated patients carrying different mutations of exon 2 presented unilateral Best VMD (CT07 [p.T4A, novel mutation] and CT11 [p.R25W] from FAMILY CT IV and FAMILY CT V, respectively). Patient CT03 (FAMILY CTII), heterozygous for the p.I230T novel mutation, and patient FG08 (FAMILY FG III), homozygous or hemizygous for the p.R92G mutation, presented bilateral multifocal Best VMD features on fundus examination.

**Figure 5:**
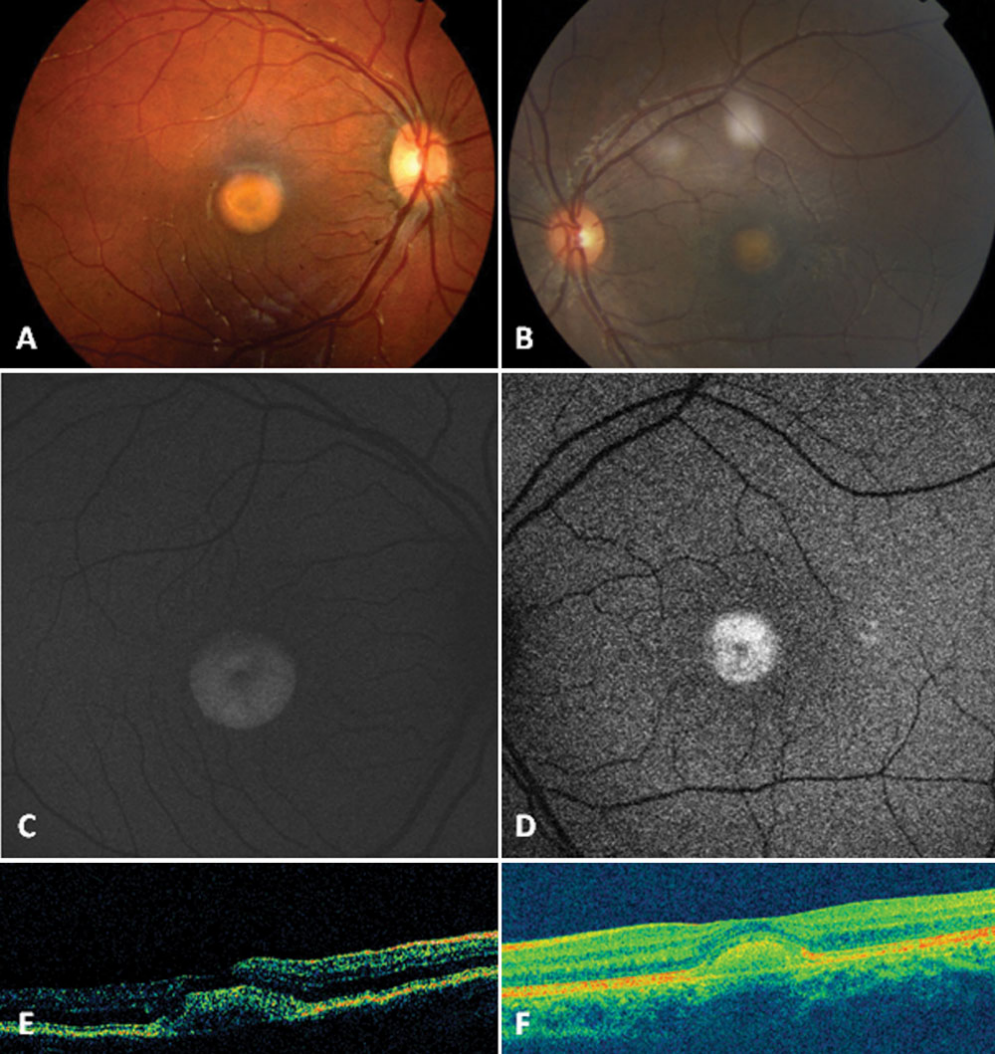
Color fundus photographs, fundus autofluorescence frames and optical coherence tomography scans of patient FG07 and patient FG09. Color fundus photographs shows typical vitelliform lesions within the macula of patient FG07 (**A**) and patient FG09 (**B**). These vitelliform lesions appear highly autofluorescent on fundus autofluorescence (**C**, patient FG07; **D**, patient FG09), and as hyper-reflective dome-shaped lesions located between the hyporeflective outer nuclear layer and the hyper reflective retinal pigment epithelium layer, on both time domain optical coherence tomography (**E**, patient FG07) and spectral domain high-definition optical coherence tomography (**F**, patient FG09) scans.

**Figure 6:**
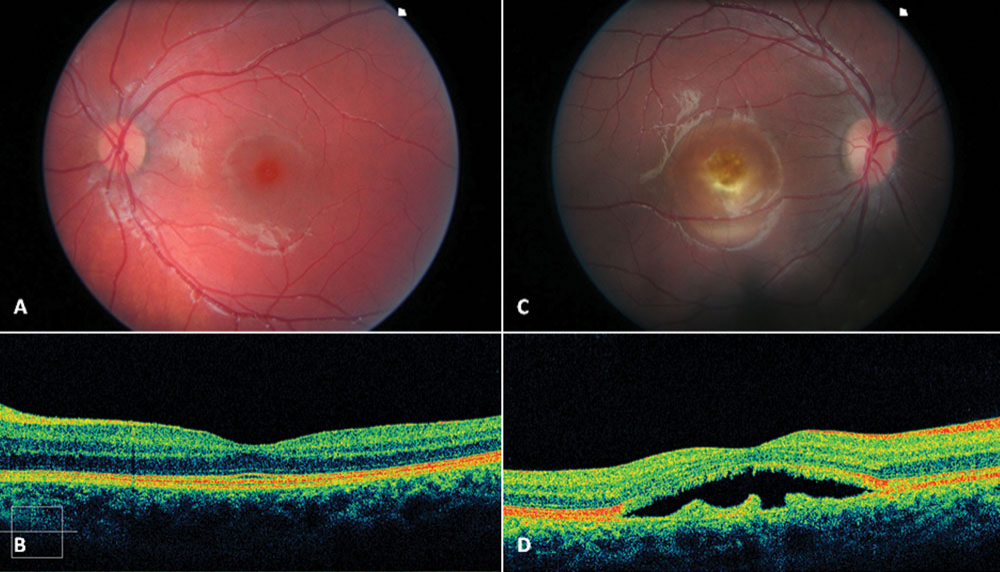
Color fundus photographs and spectral domain optical coherence tomography scans of patient CT02 and patient CT04. A normal fovea and a vitelliruptive macular lesion are shown on color fundus photographs of patient CT02 (**A**) and patient CT04 (**B**), respectively. Spectral domain high-definition optical coherence tomography scan shows, in the macular area of patient CT02, a thickening of the layer corresponding to the junction between the retinal pigment epithelium (RPE) and the interface of the inner segment and outer segment of the photoreceptor (**C**). An optically empty lesion between the RPE and the inner segment /outer segment interface, with clumping of hyper-reflective material on the posterior retinal surface and, on some parts, a hyper-reflective mottling stuck on the RPE layer (**D**), appears on the macular scan of patient CT04.

**Figure 7:**
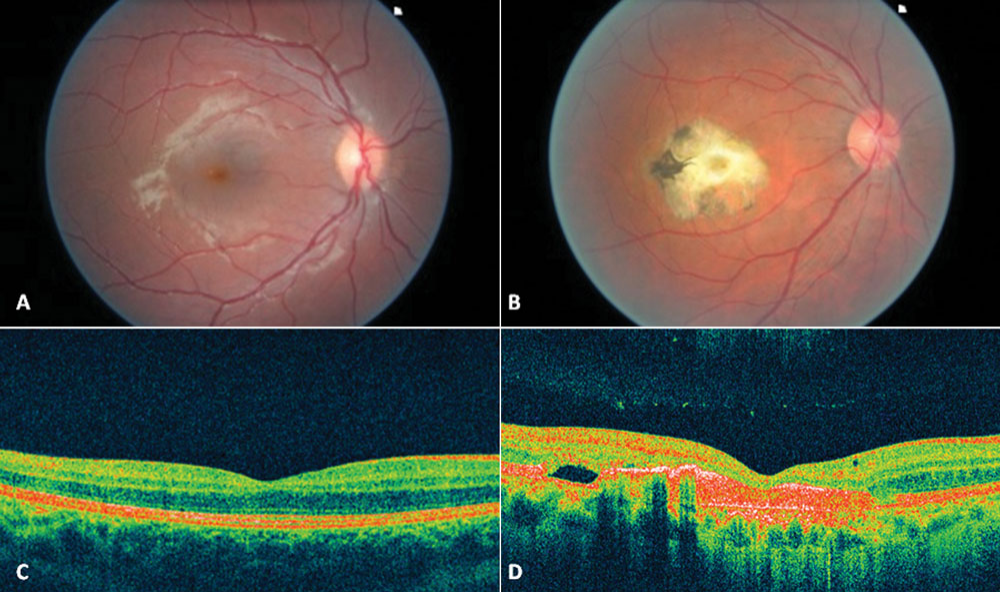
Color fundus photographs and spectral domain optical coherence tomography scans of patient CT13 and patient CT12. A normal fovea and an atrophic macular lesion are shown on color fundus photographs of patient CT13 (**A**) and patient CT04 (**B**), respectively. Spectral domain high-definition optical coherence tomography scan shows, in the macular area of patient CT13, a thickening of the layer corresponding to the junction between the retinal pigment epithelium (RPE) and the interface of the inner segment and outer segment of the photoreceptor (**C**). A thinning of all the retinal layers with enhancement of reflectivity of RPE, which seems to spread far behind it (**D**), appears on the macular scan of patients CT12.

**Figure 8:**
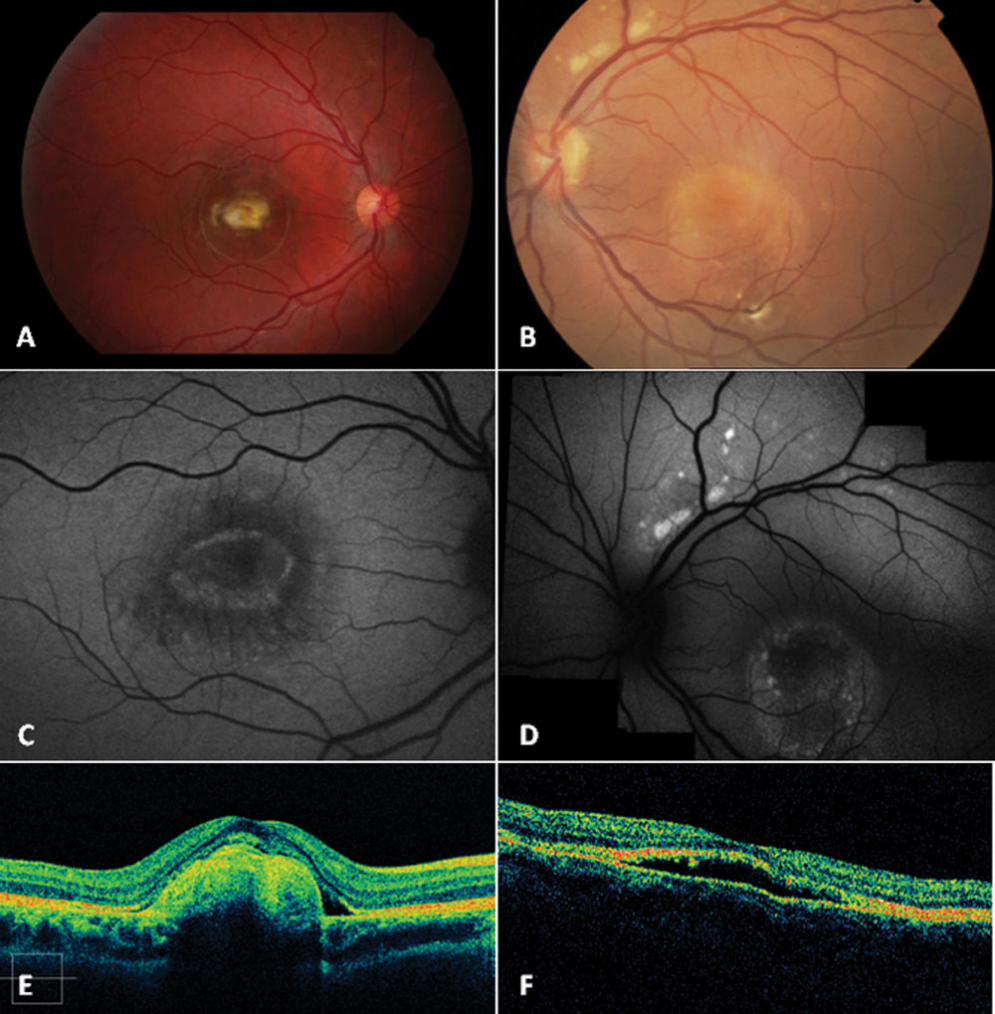
Color fundus photographs, fundus autofluorescence frames and spectral-domain optical coherence tomography scans of patient CT01 and patient FG08. Color fundus photographs show the fibrotic lesion of patient CT01 (**A**), characterized by an aspect of macular fibrosis without any detectable active choroidal neovascularization, and the multifocal vitelliform lesions of patient FG08 (**B**), characterized by a vitelliruptive aspect within the macular area. The fibrotic lesion of patient CT01 is responsible for reduced autofluorescence within the macula, on fundus autofluorescence frames (**C**), as well as for a prominent hyper reflective thickening at the level of the retinal pigment epithelium (RPE) inducing marked anterior bulging, accompanied by thinning of the sensory retina, on spectral domain high-definition optical coherence tomography scan (**E**); the multifocal vitelliform lesions of patient FG08 are visualized as multiple hyperautofluorescent lesions, on fundus autofluorescence frames, as well as an optically empty lesion between the RPE and the inner segment/outer segment interface, with clumping of hyper-reflective material on the posterior retinal surface, on time domain optical coherence tomography scan (**F**).

No association existed between the specific nature of *BEST1* mutations and expressivity in relation to age, BCVA, and stage of the disease, as evaluated by FAF, FA, and OCT (p>0.05). Mean BCVA impairment showed a statistically significant correlation to a more advanced stage of the disease (p<0.001), which was independent of patients’ age. Patient FG07 (FAMILY FG II) and patient FG09 (FAMILY FG IV) presented noticeable functional and clinical data in that they were diagnosed at an early age—about 2 years of age with typical vitelliform lesions already visible in funduscopic examination (Figure 5). Moreover, patient FG10 from the same family as patient FG09 (FAMILY FG IV) and harboring the same heterozygous *BEST1* mutation, showed normal fundus findings, OCT, and EOG at the age of 30 years. FAMILY FG01 presented noticeable functional and clinical data in that one family member (patient FG03) was diagnosed at a late age (67 years), and two family members with the same heterozygous *BEST1* mutation p.A243V (patient FG04 and patient FG05) showed normal fundus findings, OCT, and EOG at the age of 13 and 17 years, respectively. Two unrelated cases presented with the previtelliform stage as diagnosed by fundus examination, EOG, and OCT [14] (patient CT02, Family CT II and patient CT13, Family CT VI; Figure 6 and Figure 7). One case (patient CT03, FAMILY CT II) presented with the multifocal Best VMD features. The other multifocal Best VMD case (patient FG08) carried a homozygous *BEST1* mutation (exon 4 p. R92C), the same as the heterozygous one found in patient CT01 (Figure 8). Patient CT07 from FAMILY CT IV (novel mutations, p.T4A) presented an end-stage disease in one eye and no evidence of the disease in the other eye.

## Discussion

The most determinant symptom of Best VMD is the abnormal EOG with a reduced light-peak to dark-trough ratio combined with a normal ERG [19,20]. Bestrophin 1, the 585-amino acid protein encoded by the *BEST1* gene [3], is a member of the RFP-TM family of proteins, so named for their highly conserved arginine, phenylalanine, proline motif [3,21,22], which appears to exhibit properties of Ca^2+^-activated Cl^−^ channels [4,23,24]. Bestrophin 1 does not appear to be the channel itself but to act as a modulating subunit; thus channel function would directly correlate to the involved mutation. The apparent role of bestrophin 1 in the regulation of ion transport obviously affects the light peak on EOG; it is unlikely that the light peak defect itself is the cause of vision loss in Best VMD. Any connection between the light-peak deficit in Best VMD and lipofuscin accumulation in the RPE (the most common histopathologic finding in Best VMD) is speculative. However, given that ion transport is a requirement for acidification of phagolysosomal compartments and Ca^2+^ is a critical regulator of vesicle fusion, either of the proposed functions of bestrophin 1, if impaired, could lead to the accumulation of lipofuscin (and ultimately cause vision loss from lipofuscin toxicity to photoreceptors). To date more than 108 different BEST1 mutations have been reported (see the Human Gene Mutation Database).

Here, we report three novel missense changes absent from 192 control chromosomes. All three affected residues are conserved through evolution and were predicted by a structure homology-based method to have an impact on the protein (Figure 3). Two out of the three mutations occur in exon 2 (p.T4A and p.G15D), which is located in the NH_2_ cytoplasmic domain of the protein. Interestingly, it has been demonstrated that the p.T6P and p.A10V mutations that affect this domain produce currents with an amplitude >20% that of wild-type bestrophin [25]. The novel mutations p.T4A and p.G15D are predicted to change the polarity and/or the charge of the NH_2_ terminus of the protein and therefore may be regarded as disease causing. The third novel mutation, p.I230T, may alter the structure of the protein as it changes a hydrophobic residue located in the transmembrane domain of the protein into a polar residue.

Of note, the p.V9A change had previously been classified as a change of uncertain pathogenicity seemingly conservative by Petrukhin et al. [3]. In our study this change was regarded as a disease-causing mutation by virtue of its absence from 192 control chromosomes and its Polyphen PSIC score (1.949), which suggests that it may have a functional impact.

Heterozygous mutations in *BEST1*, which usually cause typical Best VMD, may also cause adult vitelliform macular degeneration [26,27], autosomal dominant bestrophinopathy, and a rare and unique condition called autosomal dominant vitreoretinochoroidopathy [28]. Burgess et al. [29] recently reported on compound heterozygous or homozygous mutations in the *BEST1* gene as the causative mutations for a distinctive retinopathy, which they named autosomal-recessive bestrophinopathy (ARB). Given that the different diseases caused by *BEST1* gene mutations may share common clinical findings, a complete clinical examination of Best VMD patients combined with molecular genetics studies of the *BEST1* gene is mandatory for adequate counseling of the families. Interestingly, while in nine of the ten pedigrees reported here the disease segregated as an autosomal dominant trait, in one family the affected patient (FG08) was apparently homozygous for a *BEST1* mutation [p.R92C]. The common origin of the patient’s parents’ homozygosity for the mutation is the likely reason, although hemizygosity at the *BEST1* locus cannot be excluded. In any case, mutational biallelism raises the question as to whether, instead of Best VMD, the patient may be affected with the autosomal-recessive bestrophinopathy described by Burgess et al. as a null phenotype of bestrophin-1 in humans [29]. Clinical examination showed that the patient had no ARB-associated, scattered, punctate flecks and retinal edema but presented with bilateral multifocal lesions consistent with the diagnosis of multifocal Best VMD. This phenotype may be considered more severe than that of another patient heterozygous for the p.R92C mutation who is affected with bilateral focal lesions complicated by CNV. This observation differs from that of Bakall et al. who reported on the histopathology of a donor eye from an individual homozygous for the *BEST1* p.W93C mutation and concluded that the clinical and pathological effects of homozygosity for the p.W93C mutation are not more severe than those reported for heterozygotes [30].

In our series we report a large interfamilial and intrafamilial clinical variability in terms of age of onset, disease progression, stage of the lesions, and visual function. We found no association between *BEST1* mutations and expressivity, with respect to age, BCVA, and stage of the disease as evaluated by FAF, FA, and OCT. Mean BCVA impairment showed a statistically significant correlation to a more advanced stage of the disease. This association was independent of the patients’ age. These data suggest that a functional impairment in Best VMD may be related to the progression of the disease rather than to a patient’s age. However, in the current series there was only one family to illustrate the phenotype of each mutation except for one mutation. This probably represents a major limitation of any statistical analysis in proposing a severity scaling.

Interestingly, the p.A243V mutation was found to be associated with late onset in one family of our Best VMD series. This finding is consistent with a previous report of a mild and relatively invariable Best VMD phenotype associated with this mutation [31]. Even though our study was not designed to investigate disease progression, the absence of phenotype in two siblings of the same family harboring the mutation may be explained by their young age (13 and 17 years). However, it is possible that these two individuals may remain unaffected (normal fundus findings, OCT, and EOG) through their life span as well. Incomplete penetrance is indeed a well known feature in BEST1 disease. Functional and clinical data in our series may support this notion. The heterozygous p.R92G an p.G15D mutations resulted in the earliest disease manifestation (at 2 years of age); however, the same mutation was also responsible for either a later onset (at the age of 11 years for FG06 [p.R92G]) or even no disease manifestation (at the age of 30 years for FG10 [p.G15D]) within the same families (FAMILY FG II and FAMILY FG IV, respectively).

All patients except two had bilateral macular lesions. Two patients presented with unilateral disease, but this could not be related either to their age or to their genotype. Indeed, one of them, aged 27 (CT07), shared the p.T4A mutation with his 23-year-old sibling presenting with bilateral lesions (CT08). Similarly, the second patient, a 70-year-old man (CT11) carried the p.R25W mutation responsible for bilateral lesions in two of his young relatives, aged 10 and 36. Similarly, CNV did not appear to correlate with the mutation, as suggested by the intrafamilial variability of this trait.

Bilateral multifocal Best VMD lesions were diagnosed at the age of 41 in a patient heterozygous for another *BEST1* mutation, p.I230T. Two younger relatives (aged 9 and 11) presented with an early-stage lesion; progression is uncertain.

The wide variability of clinical expression of *BEST1* mutations within and between families is consistent with previous reports [27,32-40]. Owing to this wide variability of clinical expression, it is difficult to compare our findings with other previously published series. Moreover, we adopted a widely accepted clinical classification, and, based on fundus biomicroscopy, all eyes were graded as showing only one of the progressive stages of Best VMD; thus, for example, in contrast with Boon et al. [30], we did not consider characteristics attributable to different stages. However, the clinical features reported here for each progressive stage were typical and actually consistent with other Best VMD series.

One limitation of the current study was the absence of real co-segregation analysis for the families with novel reported changes. Another limitation was that we did not perform, systematically, ERG in our patients and thus we were not able to distinguish whether an abnormal light rise on EOG would have been due to either photoreceptor or RPE dysfunction.

Overall in our series, particularities were found in two affected patients showing unilateral Best VMD, in two affected patients showing, in both eyes, multifocal Best VMD, and in four affected patients (six eyes) who were treated for CNV. All these are well known possible features of Best VMD. Three out of 23 patients (13%) with the *BEST1* mutation showed normal fundus, OCT, and EOG findings.

In conclusion, variability of clinical expression of *BEST1* mutations suggests that cis or trans-acting genetic modifiers may modulate the functional and clinical data.
